# Prolyl carboxypeptidase activity in the circulation and its correlation with body weight and adipose tissue in lean and obese subjects

**DOI:** 10.1371/journal.pone.0197603

**Published:** 2018-05-17

**Authors:** Kaat Kehoe, Heidi Noels, Wendy Theelen, Emilie De Hert, Shenguan Xu, An Verrijken, Thierry Arnould, Erik Fransen, Nina Hermans, Anne-Marie Lambeir, Per Venge, Luc Van Gaal, Ingrid De Meester

**Affiliations:** 1 Laboratory of Medical Biochemistry, Department of Pharmaceutical Sciences, University of Antwerp, Antwerp, Belgium; 2 Institute for Molecular Cardiovascular Research, RWTH Aachen University, Aachen, Germany; 3 Section of Clinical Chemistry, Department of Medical Sciences, University of Uppsala, Uppsala, Sweden; 4 Department of Endocrinology, Diabetology and Metabolism, Antwerp University Hospital, Edegem, Belgium; 5 Laboratory of Experimental Medicine and Paediatrics (LEMP), Faculty of Medicine and Health Sciences, University of Antwerp, Antwerp, Belgium; 6 Laboratory of Biochemistry and Cell Biology (URBC), Namur Research Institute for Life Sciences (NARILIS), University of Namur (UNamur), Namur, Belgium; 7 StatUa Center for Statistics, University of Antwerp, Antwerp, Belgium; 8 Natural Products & Food Research and Analysis (NatuRA), Department of Pharmaceutical Sciences, University of Antwerp, Antwerp, Belgium; Medical University of Vienna, AUSTRIA

## Abstract

**Background:**

Prolyl carboxypeptidase (PRCP) is involved in the regulation of body weight, likely by hydrolysing alpha-melanocyte-stimulating hormone and apelin in the hypothalamus and in the periphery. A link between PRCP protein concentrations in plasma and metabolic disorders has been reported. In this study, we investigated the distribution of circulating PRCP activity and assessed its relation with body weight and adipose tissue in obese patients and patients who significantly lost weight.

**Methods:**

PRCP activity was measured using reversed-phase high-performance liquid chromatography in different isolated blood fractions and primary human cells to investigate the distribution of circulating PRCP. PRCP activity was measured in serum of individuals (n = 75) categorized based on their body mass index (BMI < 25.0; 25.0–29.9; 30.0–39.9; ≥ 40.0 kg/m^2^) and the diagnosis of metabolic syndrome. Differences in serum PRCP activity were determined before and six months after weight loss, either by diet (n = 45) or by bariatric surgery (n = 24). Potential correlations between serum PRCP activity and several metabolic and biochemical parameters were assessed. Additionally, plasma PRCP concentrations were quantified using a sensitive ELISA in the bariatric surgery group.

**Results:**

White blood cells and plasma contributed the most to circulating PRCP activity. Serum PRCP activity in lean subjects was 0.83 ± 0.04 U/L and increased significantly with a rising BMI (p<0.001) and decreased upon weight loss (diet, p<0.05; bariatric surgery, p<0.001). The serum PRCP activity alteration reflected body weight changes and was found to be positively correlated with several metabolic parameters, including: total, abdominal and visceral adipose tissue. Plasma PRCP concentration was found to be significantly correlated to serum PRCP activity (0.865; p<0.001). Additionally, a significant decrease (p<0.001) in plasma PRCP protein concentration (mean ± SD) before (18.2 ± 3.7 ng/mL) and 6 months after bariatric surgery (15.7 ± 2.7 ng/mL) was found.

**Conclusion:**

Our novel findings demonstrate that white blood cells and plasma contributed the most to circulating PRCP activity. Additionally, we have shown that there were significant correlations between serum PRCP activity and various metabolic parameters, and that plasma PRCP concentration was significantly correlated to serum PRCP activity. These novel findings on PRCP activity in serum support further investigation of its *in vivo* role and involvement in several metabolic diseases.

## Introduction

Prolyl carboxypeptidase (PRCP, EC 3.4.16.2), a lysosomal carboxypeptidase, changes the biological activity of α-melanocyte-stimulating hormone (α-MSH) [1], as well as angiotensin II and III [2] and des-Arg^9^-bradykinin [3] by cleaving off one C-terminal amino acid when alanine or proline are in the penultimate position [4]. Previous reports have suggested an important role for PRCP in the regulation of food intake and body weight by truncation of α-MSH [1]. This 13 amino-acid peptide (referred to as α-MSH 1–13) is a critical anorexigenic neuromodulator in the hypothalamus that suppresses appetite and inhibits food intake by binding target neurons expressing the melanocortin receptors 3 (MC3R) and 4 (MC4R) [5]. Little was known about the inactivation of α-MSH 1–13, until Wallingford *et al*. discovered that PRCP is responsible for removing the C-terminal Val of α-MSH 1–13, producing α-MSH 1–12, which does not trigger the MCR and thus is not neuroactive [1].

Furthermore, PRCP knockout (KO) mice are known to have a significantly lower body weight, total fat, BMI and body length, compared to wild-type mice. PRCP KO mice consume significantly less food compared to controls and have been shown to be resistant to diet-induced obesity [1]. Pharmacological inhibition of PRCP, centrally as well as solely in the periphery, decreases food intake in wild-type and obese mice [6,7]. In addition, our group has shown that pyroglutamated apelin-13, an adipokine mostly known for its cardiovascular effects, is also truncated by PRCP at the C-terminus [8]. This peptide has been related to obesity and might also play an important role in PRCP’s function in body weight control [9–11].

PRCP is ubiquitously expressed but data concerning the cellular sources of plasma PRCP or PRCP in different circulating and vascular cell types are scarce [12,13]. Previous reports have indicated the presence of PRCP in the lysosomes and in the granular fraction of leukocytes [14,15]. PRCP activity has also been demonstrated in primary human fibroblasts and endothelial cells cultured from human pulmonary arteries [16]. In 1995, Jackman *et al*. provided evidence that PRCP is active in human alveolar macrophages [17]. More recently, PRCP was purified from human neutrophils, thereby revealing that these cells highly express PRCP [18]. Furthermore, it has been shown that PRCP is not only located in the lysosomes but also on the cell membrane of endothelial cells [19]. Although these data are informative, these do not permit us to predict which cell or tissue type(s) most likely contribute(s) to the circulating PRCP activity.

Plasma PRCP concentrations have been shown to be increased in obesity, diabetes, and cardiovascular dysfunction and significant correlations with several metabolic and cardiovascular parameters have been determined [18]. More recently, differences in PRCP expression and function have been observed in the heart, kidney and plasma of Zucker diabetic fatty rats fed a high-fat diet compared to Zucker lean controls. Plasma PRCP concentrations have been found to be higher in diabetic rats compared to the controls. Furthermore, PRCP protein has been found to be significantly elevated in plasma of five uncontrolled diabetic patients and treatment of these patients with anti-diabetic drugs (such as metformin) returned the PRCP protein levels almost back to baseline. However, the underlying mechanisms by which the elevated plasma levels were reversed have not been fully elucidated and require further investigation. It is possible that the decrease in PRCP protein levels is related to simultaneous body weight reduction caused by metformin therapy [20].

Despite information on gene and protein expression, the question regarding the actual PRCP activity in plasma remains unanswered. Indeed, the majority of PRCP’s functions have been assigned to its enzymatic activity and to its role in peptide turnover. These PRCP activity measurements have never been performed in well-characterized larger populations and could provide complementary information regarding PRCP’s *in vivo* function.

The aims of this study were 1) to investigate PRCP activity in different primary cell types (monocytes, macrophages, lymphocytes, granulocytes, endothelial cells) to determine the distribution of PRCP activity in the circulation and 2) to measure PRCP activity in serum collected from individuals categorized based on their BMI and the diagnosis of metabolic syndrome (MS). Additionally, differences in serum PRCP activity were determined before and after significant weight loss, either by diet or by bariatric surgery. Correlations between the serum PRCP activity and several metabolic and biochemical parameters were assessed. In addition, plasma PRCP protein concentrations were also quantified in the bariatric surgery group.

## Materials and methods

### PRCP activity in human circulation

The study was approved by the local Ethics Committee at the University Clinic of Aachen, Germany (EK191/14) and blood was collected with written consent of the blood donors. Human blood was collected from six healthy volunteers using citrate as an anticoagulant and the first 3 mL was discarded. White blood cells, blood platelets, platelet-rich and platelet-free plasma were isolated for analysis of PRCP activity.

The number of white blood cells and platelets per mL were counted using an automated hematology analyser. After adding prostacyclin (100 ng/mL), the blood sample was centrifuged for 15 min at 365 *g* without brakes. The cell pellet was used for the isolation of white blood cells, as described below. The supernatant, being platelet-rich plasma, was collected, supplemented with acid-citrate-dextrose (ACD) (1:6 Vol.-%) and apyrase (0.1 units/mL), and centrifuged for 10 min at 1045 *g* without brakes. The supernatant was used to prepare platelet-free plasma by centrifuging again for 10 min at 2500 *g*. Instead, the platelet-containing pellet was gently resuspended in Hepes pH 6.6 containing glucose (1 mg/mL), bovine serum albumin (BSA, 1 mg/mL), ACD (1:15 Vol.-%), apyrase (0.1 units/mL) and prostacyclin (100 ng/mL). After centrifugation for 10 min at 1045 *g* without brakes, the platelet pellet was gently resuspended in Hepes pH 7.45 containing glucose (1 mg/mL) and BSA (1 mg/mL). Isolated cells were counted using a Neubauer chamber. All fractions were frozen at -20 °C for analysis of PRCP activity.

Where indicated, isolated platelets were stimulated with thrombin (20 units/mL) in the presence of CaCl_2_ (2 mM) for 45 min at 37 °C. Control platelets were kept for 45 min at 37 °C in the presence of prostacyclin (100 ng/mL). After stimulation, platelets were centrifuged for 2 min at 3000 *g* without brakes. The supernatants as well as the platelet pellets were frozen at -20 °C for analysis of PRCP activity.

To isolate white blood cells, citrate-anticoagulated human blood was centrifuged for 15 min at 365 *g*. The cell pellet was incubated in red blood cell lysis buffer (0.155 M NH_4_Cl, 0.01 M KHCO_3_ and 0.1 mM EDTA in water) for 10 minutes at room temperature. After centrifugation for 5 min at 300 *g*, the cells were washed with phosphate buffered saline (PBS), counted and the cell pellet was frozen at -20 °C for PRCP analysis.

To calculate the relative contribution of white blood cells, platelets and platelet-free plasma to the total PRCP activity in blood, PRCP activity of white blood cell lysates, platelet lysates, platelet-free plasma as well as total blood lysates were first expressed in U/mL blood, after which the relative contribution was calculated. For white blood cells and platelets, PRCP activity in U/mL blood was calculated as U/cell number multiplied by the cell number per mL of blood. The platelet-free plasma volume fraction of total blood was calculated as “1—haematocrit value/100”, neglecting the volume of white blood cells. Next, the contribution of platelet-free plasma to total circulating PRCP activity (in U/mL blood) was calculated as “PRCP activity (U/mL plasma) x platelet-free plasma volume fraction of blood”.

### PRCP activity in peripheral blood mono- and polymorphonuclear cells

Peripheral blood mononuclear cells (PBMCs) were isolated by Ficoll-Paque density gradient centrifugation of buffy coats (Blood Transfusion Centre) [21]. Buffy coats were diluted twice in DPBS (1:1, vol/vol) (Lonza, Verviers, Belgium) and layered on top of the Ficoll-Paque PREMIUM (Sigma-Aldrich, Diegem, Belgium) solution and centrifuged for 40 min at 400 *g* (no brakes). PBMCs were collected from the interface and subsequently washed two times with complete RPMI 1640 medium (Life Technologies, Ledeberg, Belgium). PBMCs of one buffy coat were grown overnight in complete RPMI 1640 medium in four T25 culture flasks at 37 °C under 5% CO_2_. On day 2, the non-adherent cells (predominantly lymphocytes) were collected. The adherent cells (mostly monocytes) were either harvested and collected or differentiated into M1 and M2 macrophages for 6 days in complete RPMI medium supplemented with 100 ng/mL granulocyte macrophage colony stimulating factor (Immunotools, Friesoythe, Germany) or 20 ng/mL macrophage colony stimulating factor (Immunotools) according to our in-house protocol [22]. Macrophages were harvested or further activated by incubation of the cells with 20 ng/mL lipopolysaccharide (LPS, Sigma-Aldrich) and 100 U/mL interferon-γ (IFN-γ, Immunotools) for 24 h for M1 activation and with 20 ng/mL interleukin-4 (Immunotools) for M2 activation. To confirm appropriate macrophage activation by these stimulation protocols, we determined tumor necrosis factor-α (TNFα) levels in the 8 h aspirate and interleukin-6 (IL-6), interleukin-1β (IL-1β) and interleukin-10 (IL-10) levels in the 24 h aspirate by using ELISA. All ELISA’s were performed according to the manufacturer’s instructions (Immunotools) [22].

Subsets of lymphocytes (CD4^+^ T cells, CD8^+^ T cells and CD19^+^ B cells) were isolated from PBMCs using Dynabeads FlowComp^™^ Human CD4, CD8 and CD19 pan B (Life Technologies), according to manufacturer’s instructions.

Granulocytes were isolated from the lower fraction of the Ficoll-Paque centrifugation. A 6% dextran (Sigma-Aldrich) sedimentation and red blood cell lysis was performed to obtain a pure granulocyte population. According to Maqbool *et al*. > 95% of the isolated cells are neutrophils [23]. For confirmation, hematoxylin and eosin staining was performed. Next, granulocytes were activated by incubating the cells for 30 min with 10 μM phorbol 12-myristate 13-acetate (PMA, Sigma-Aldrich) at 37 °C under 5% CO_2_. Supernatants were collected where relevant and all cells described above were lysed for PRCP activity measurement (S1 Appendix).

### Human aortic endothelial and smooth muscle cells

Human aortic endothelial (EC) cells and human aortic smooth muscle (SMC) cells (both from Promocell, Heidelberg, Germany) were cultured in Endothelial Cell culture Medium MV, respectively, Smooth Muscle Cell growth medium 2 (Promocell) according to the manufacturer’s protocol. Cells were plated in 6 well plates and stimulated with 10 ng/mL TNFα for 24 h. Supernatants were collected and the cells were lysed for PRCP activity measurements.

### Cell lysates

Washed cells were suspended in a lysis buffer for PRCP activity (1% octylglucoside, 10 mM EDTA, 70 μg/mL aprotinin in a 50 mM Tris buffer, pH 8.3). Suspensions were then incubated on ice for 1 h at 4 °C and centrifuged for 10 min at 12000 *g* at 4 °C. The supernatant was withheld and stored at -20 °C until further analysis. The protein content of the cell lysates was determined according to the Bradford method using bovine serum albumin (Sigma-Aldrich) as a standard [24].

### PRCP activity and protein measurements

PRCP activity was measured using a previously validated reversed-phase high-performance liquid chromatography assay [25]. The hydrolysis of the substrate N-benzyloxycarbonyl-L-Pro-Phe (Z-Pro-Phe, Bachem, Bubendorf, Switzerland) by PRCP at 37 °C leads to the release of Z-Pro and Phe. The quantification of the peak height of either of these two products is proportional to the PRCP activity, which is expressed as units per liter (U/L). One unit defines the amount of enzyme needed to hydrolyse 1 μmol of substrate per minute. A sensitive in-house polyclonal antibody-based ELISA was used for the measurement of PRCP protein concentrations in plasma [18].

### Study populations

Patients visiting the obesity clinic of the Antwerp University Hospital (a tertiary referral facility) for a problem of overweight or obesity, presenting at their own initiative or referred by their treating physician, were consecutively recruited. Every patient underwent a standard metabolic work-up approved by the Ethics Committee of the Antwerp University Hospital (reference 6/25/125, Belgian registration number B30020071389) and requiring written informed consent of the patient. All patients were female and predominantly of Western European descent. At enrolment none were involved in a weight management program. Subjects had to be 18 years or older. PRCP activity was first measured in baseline serum of female adults categorized based on their BMI (< 25.0; 25.0–29.9; 30.0–39.9; ≥ 40.0 kg/m^2^) and the diagnosis of metabolic syndrome according to Alberti *et al* [26]. 10–15 patients were included per group (study cohort 1). Serum PRCP activity was also investigated in female adults before and 6 months after weight loss either by diet (n = 45) or by bariatric surgery (n = 24) (study cohort 2). Population characteristics are summarized in Tables 1 and 2. In addition, serum PRCP activity was correlated with several metabolic and biochemical parameters and with plasma PRCP protein concentration. See S2 Appendix for individual data points and more information about the inclusion criteria of the patients and metabolic work-up.

**Table 1:** Patient characteristics of study cohort 1.

	BMI < 25	BMI 25–29.9		BMI 30–39.9		BMI ≥ 40	
	MS-	MS-	MS+	MS-	MS+	MS-	MS-
**N**	15	10	10	10	10	10	10
**Age (years)**	37.3 ± 9.9	41.0 ± 17.4	44.1 ± 11.8	43.4 ± 11.0	42.3 ± 15.2	35.8 ± 12.4	43.4 ± 16.5
**Height (m)**	1.66 ± 0.06	1.66 ± 0.08	1.65 ± 0.05	1.66 ± 0.03	1.68 ± 0.1	1.67 ± 0.06	1.62 ± 0.05
**Weight (kg)**	59.0 ± 4.9	76.7 ± 8.3	78.2 ± 4.3	95.1 ± 8.1	99.4 ± 14.9	120.3 ± 12.7	119.3 ± 11.9
**BMI (kg/m**^**2**^**)**	21.4 ± 1.5	27.7 ± 1.1	28.3 ± 1.3	34.7 ± 2.9	35.1 ± 1.8	43.3 ± 3.2	45.5 ± 3.5

Mean value ± SD is shown for all parameters, except N (absolute number). MS-/+: diagnosis of the metabolic syndrome no (-), yes (+)

**Table 2:** Patient characteristics of study cohort 2.

	Bariatric surgery		Diet	
	Before	After	Before	After
**N**	24		45	
**Age (years)**	38.7 ± 12.6		46.0 ± 12.8	
**Height (m)**	1.64 ± 0.05		1.65 ± 0.06	
**Weight (kg)**	114.6 ± 17.6	88.1 ± 15.2	96.2 ± 16.5	86.2 ± 16.2
**BMI (kg/m**^**2**^**)**	42.5 ± 6.1	32.7 ± 5.3	35.3 ± 4.9	31.6 ± 4.8

Mean value ± SD is shown for all parameters, except N (absolute number).

### Statistical analysis

Data were analysed using the statistical SPSS software version 22 (IBM, New York, United States). Differences in PRCP activity between the various blood fractions collected from six healthy volunteers were analysed with Friedman and Wilcoxon Signed-Rank test. Differences in PRCP activity between various cell types and differences in cytokine levels in the supernatant of monocytes and (activated) M1 macrophages were analysed with Kruskal-Wallis and Mann-Whitney U-tests. P-values were corrected for multiple testing based on scientifically relevant pairwise comparisons. A one-way ANOVA, followed by a post-hoc analysis, using Tukey’s HSD correction for multiple comparisons, was carried out to assess differences in serum PRCP activity between the different BMI categories. An ANCOVA was used to assess the influence of both BMI and metabolic syndrome on the PRCP activity in serum. A t-test for paired samples was used to compare the PRCP activity and protein concentration before and after weight loss either by diet or bariatric surgery. The relation between the PRCP activity and several metabolic and biochemical parameters and PRCP protein concentration was described using Pearson’s correlation coefficient. Both baseline levels as well as changes after weight loss (indicated by Δdelta) were investigated. PRCP activity levels are presented as boxplots or as median with interquartile range (GraphPad Prism 7 software, La Jolla, California, USA). Significant changes are indicated with an asterisk (*p<0.05, **p<0.01, ***p<0.001).

## Results

### PRCP activity in human circulation

**Fig 1:**
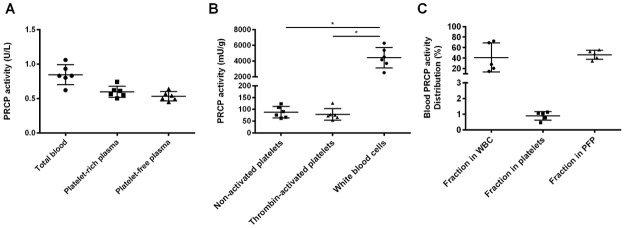
PRCP activity in human circulation. (A) PRCP activity was measured in total lysed blood, platelet-rich and platelet-free plasma but no significant changes were found (p = 0.174). (B) PRCP activity was significantly higher in lysed white blood cells compared to resting and thrombin-activated blood platelets (p = 0.028). (C) PRCP activity measured in human blood is mainly attributed to white blood cells and the plasma fraction. (*p<0.05, n = 4–6 per group).

PRCP activity (median with interquartile range) was measured in total lysed blood (12.3 [10.5–13.5] mU/g), platelet-rich (10.3 [9.1–11.4] mU/g) and platelet-free (9.8 [8.9–11.1] mU/g) plasma of 6 healthy donors. As shown in Fig 1A, no significant differences were observed between these groups, indicating that there is little to no PRCP activity found in platelets (p = 0.174). PRCP was also measured in isolated white blood cells, resting platelets and platelets activated with thrombin. Again, low PRCP activity was found in resting platelets and thrombin activation had no effect on the enzyme activity. The specific activity measured in white blood cells was approximately fifty times higher compared to platelets (p = 0.028, Fig 1B). When comparing the relative contribution of blood fractions to the circulating PRCP activity, white blood cells and the platelet-free plasma fraction contributed for 41%, respectively, 46% to the total PRCP activity in blood (Fig 1C). Overall, white blood cells and the plasma fraction contribute most to the circulating PRCP activity.

### PRCP activity in primary human cells

**Fig 2:**
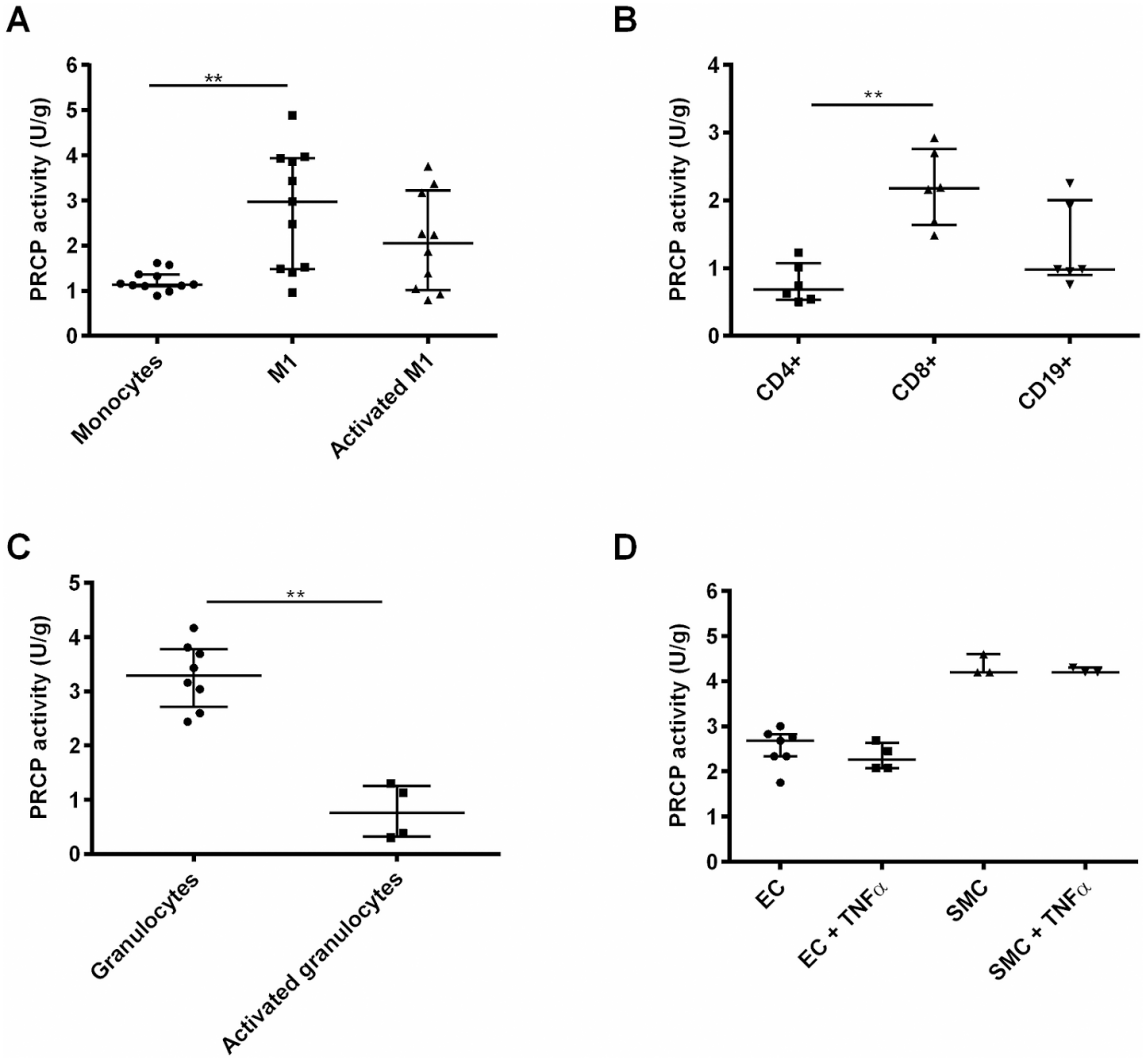
PRCP activity in primary human cells. (A) PRCP activity was significantly increased upon monocyte-to-macrophage (M1 phenotype) differentiation. (B) PRCP activity was measured in different subsets of lymphocytes and was found highest in CD8^+^ cytotoxic T cells. (C) PRCP activity was measured in resting and PMA-activated granulocytes and (D) in control and stimulated (TNFα, 24h) human aortic endothelial and smooth muscle cells. (**p<0.01, n = 3–11 per group).

Appropriate activation of M1 macrophages was confirmed by ELISA, since the levels of TNFα, IL-1β and IL-6 were significantly upregulated in the supernatant of activated M1 macrophages (p<0.05, data not shown). PRCP activity is significantly higher in resting M1 macrophages (p = 0.003) compared to monocytes. Even when we take into account that freshly isolated monocytes would be a more suitable comparator as the adhesion to plastic and overnight culture could already alter the monocyte phenotype, these results clearly show that an increase in PRCP activity was found upon monocyte-to-M1-macrophage differentiation. M2 macrophage activation could not be confirmed, since no clear upregulation of IL-10 could be measured in the supernatant of activated M2 macrophages. Therefore, no conclusions could be drawn concerning the M2 macrophages. Furthermore, a decrease in PRCP activity was observed after PMA stimulation of granulocytes (p = 0.007). The PRCP activity is higher in CD8^+^ cytotoxic T cells compared to CD4^+^ helper T cells. Human aortic endothelial and smooth muscle cells were stimulated with TNFα for 24 h. However, no significant difference in PRCP activity was found after stimulation. The PRCP activity in human aortic smooth muscle cells is higher than in the human aortic endothelial cells. The results are all shown in Fig 2. In addition to human cells, we also isolated and cultured primary cells from the bone marrow of wild-type mice. Similar results as for the human blood cell populations were found (S3 Appendix).

### Serum PRCP activity in obese patients

**Fig 3:**
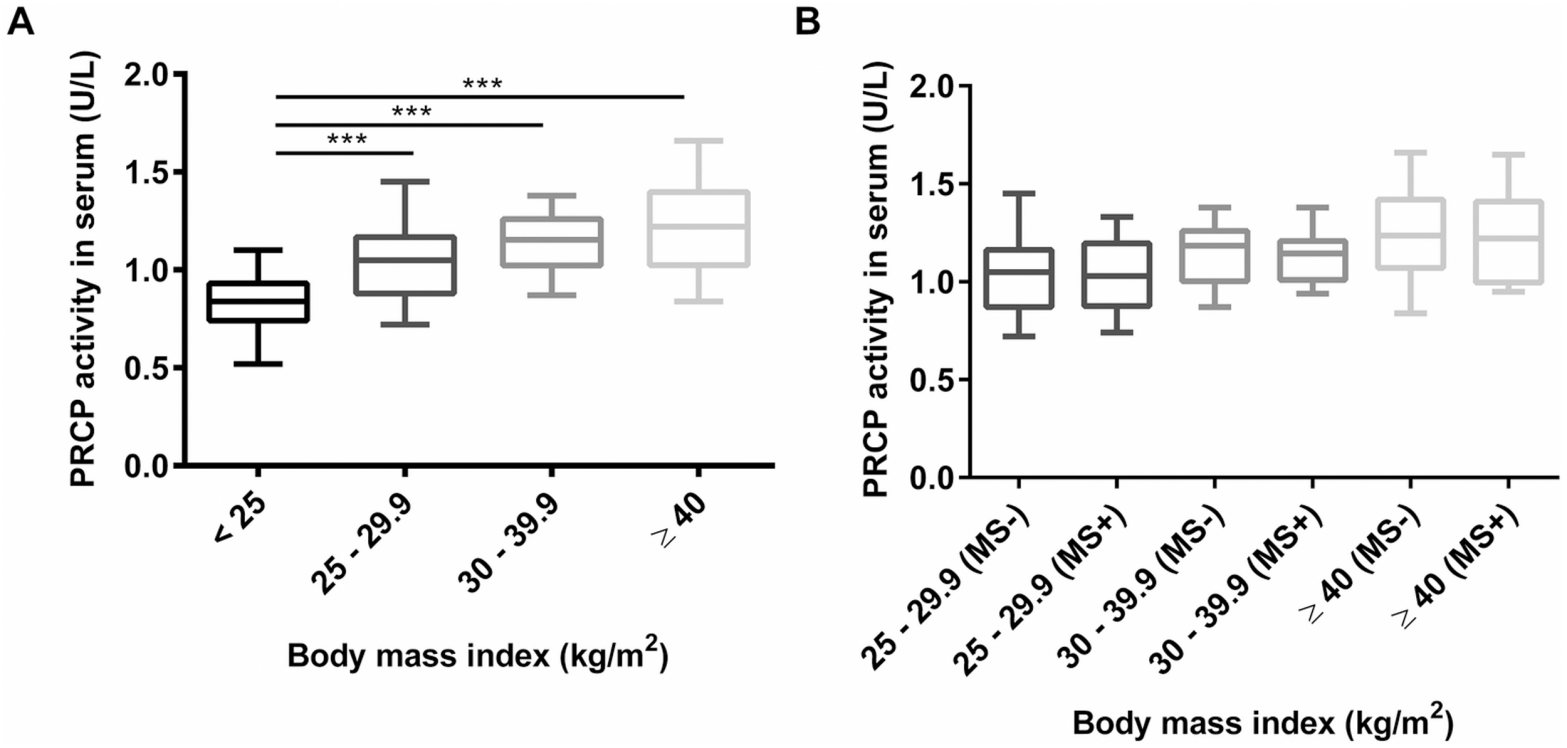
Serum PRCP activity in lean and obese patients. (A) PRCP activity was measured in serum of lean and obese patients categorized based on their BMI and (B) the diagnosis of metabolic syndrome (no (-), yes (+)). (***p<0.001, n = 10–20 per group).

**Fig 4:**
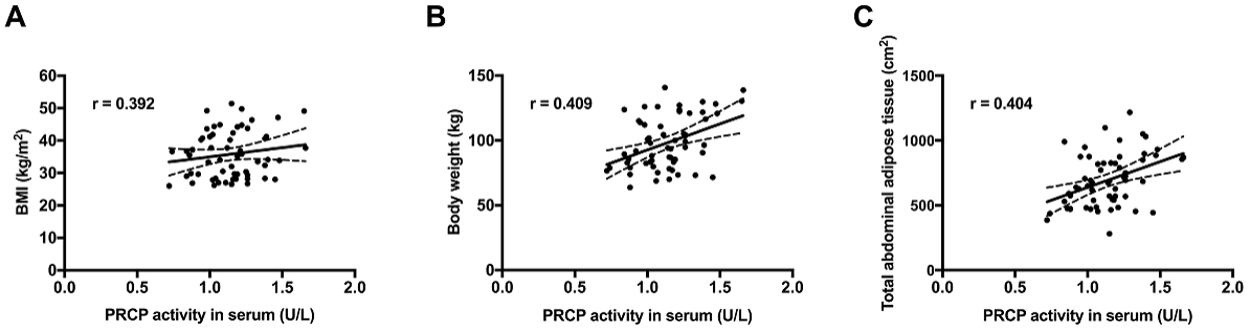
Correlations between serum PRCP activity and BMI, body weight and total abdominal adipose tissue. Correlation between serum PRCP activity and (A) BMI (p = 0.002), (B) body weight (p = 0.001) and (C) total abdominal adipose tissue (p = 0.001) in overweight/obese patients (n = 60).

PRCP activity was measured in serum of 15 lean and 60 obese female patients, which were further divided into three subgroups based on their BMI. The mean serum PRCP activity (mean ± SD) in lean subjects (BMI < 25.0 kg/m^2^) was 0.83 ± 0.04 U/L, 1.03 ± 0.04 U/L in overweight patients (BMI 25.0–29.9 kg/m^2^), 1.13 ± 0.03 U/L in obese patients (BMI 30.0–39.9 kg/m^2^) and 1.23 ± 0.05 U/L in morbidly obese patients (BMI ≥ 40.0 kg/m^2^). Thus, the PRCP activity in serum of the groups with elevated BMI is significantly elevated compared to the PRCP activity in the lean group, as shown in Fig 3A. The three subgroups were then further divided based on the diagnosis of metabolic syndrome (MS- or MS+). As presented in Fig 3B, no significant differences in mean serum PRCP activity were found between metabolic syndrome (MS+) patients and those without (MS-) (p > 0.05). In parallel, we studied PRCP activity in serum of rats with metabolic syndrome. Again, no significant differences in serum PRCP activity between the controls and rats with metabolic syndrome were found (S4 Appendix). Correlations between serum PRCP activity in overweight/obese patients (n = 60) and patient age, several metabolic and biochemical parameters were investigated. An overview of the significant correlations is given in Table 3 and correlations between serum PRCP activity and BMI, body weight and total abdominal tissue are shown in Fig 4. The correlation with the amount of adipose tissue is intriguing since this suggests that PRCP might be secreted in serum by adipocytes. No correlations were found with systolic and diastolic blood pressure, lipid parameters, percentage glycated haemoglobin A1, oral glucose tolerance test results (baseline glucose and insulin levels and glucose level at 120 min) and high-sensitive C-reactive protein levels (r < 0.25).

**Table 3:** Significant correlations between serum PRCP activity in overweight/obese patients (n = 60) and several metabolic parameters.

Parameter	Pearson correlation	p-value
**Body weight (kg)**	0.409	0.001
**BMI (kg/m**^**2**^**)**	0.392	0.002
**Waist (cm)**	0.385	0.002
**Hip (cm)**	0.360	0.005
**Visceral fat (cm**^**2**^**)**	0.376	0.003
**Subcutaneous fat (cm**^**2**^**)**	0.352	0.006
**Total abdominal fat (cm**^**2**^**)**	0.404	0.001

### Serum PRCP activity before and after weight loss

**Fig 5:**
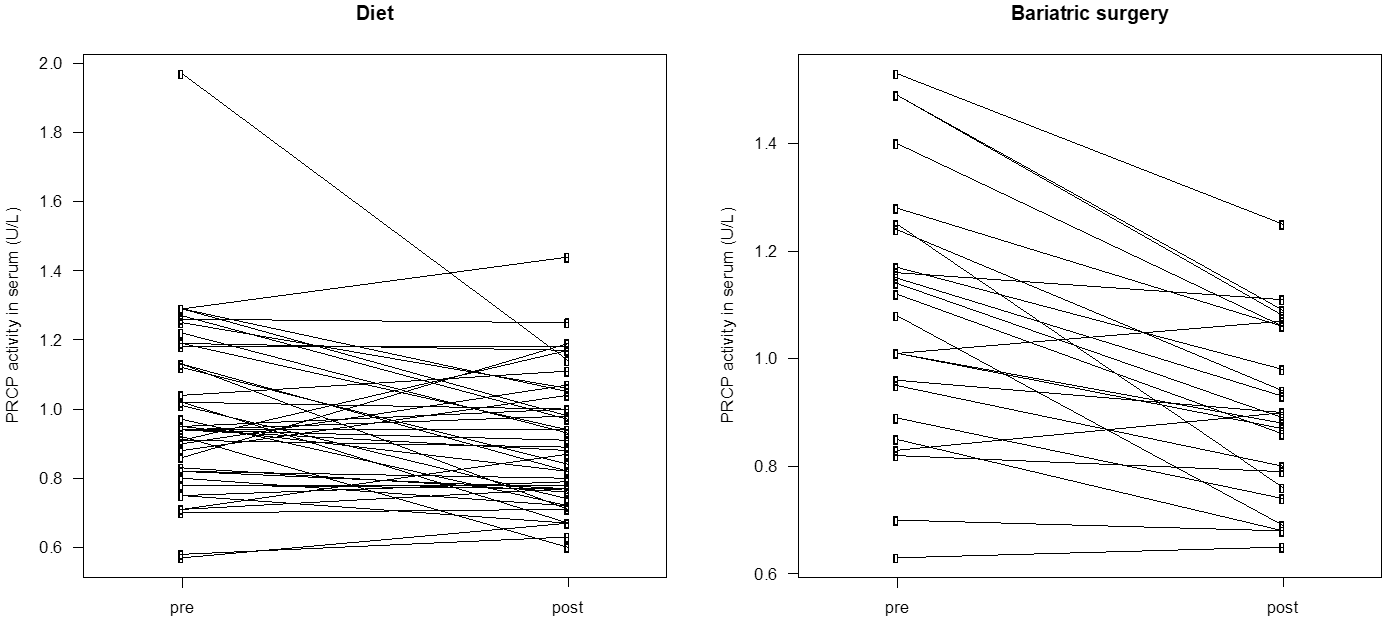
Serum PRCP activity changes after weight loss. Line plots indicating the individual change in PRCP activity in serum of patients who lost weight either by diet (n = 45, p = 0.015) or by bariatric surgery (n = 24, p < 0.001).

**Fig 6:**
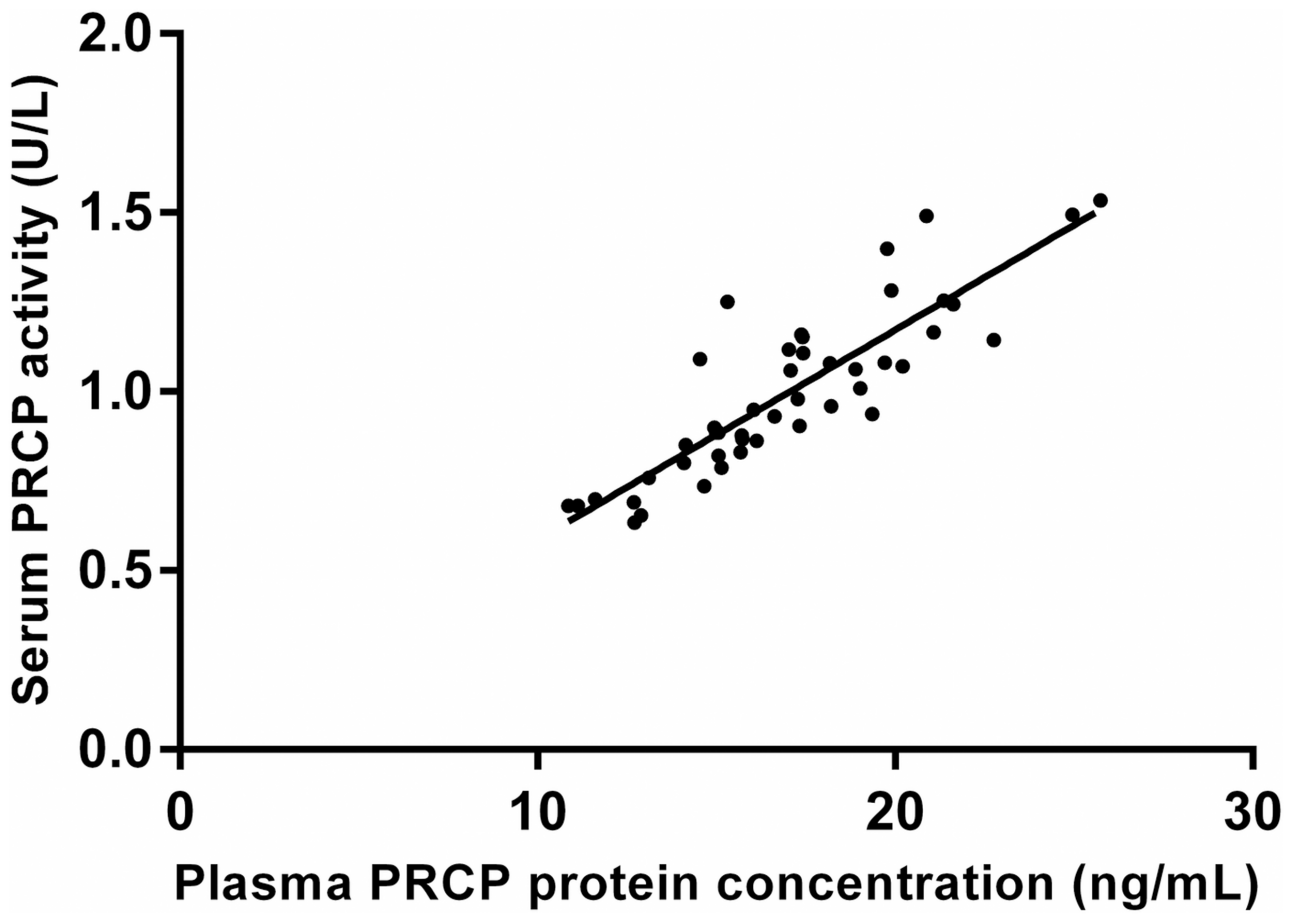
Correlation between serum PRCP activity and plasma PRCP protein concentration. Correlation between serum PRCP activity and plasma PRCP protein concentration in the bariatric surgery group (0.865; p < 0.001, n = 24).

A group of 69 obese patients were treated with either a hypocaloric diet (n = 45) or bariatric surgery (n = 24). Diet as well as bariatric surgery caused a significant decrease in body weight (mean ± SD; 10.0 ± 7.2 vs. 26.5 ± 7.9 kg, p < 0.001) and BMI (mean ± SD; 3.6 ± 2.7 vs. 9.8 ± 3.0 kg/m^2^, p < 0.001) although more pronounced after surgery. The mean serum PRCP activity (mean ± SD) before and 6 months after diet was 0.99 ± 0.25 U/L and 0.90 ± 0.25 U/L, while before and after bariatric surgery 1.09 ± 0.24 U/L and 0.90 ± 0.19 U/L. In both cases there was a significant decrease in the mean serum PRCP activity (mean ± SD; 0.08 ± 0.03 U/L for diet and 0.19 ± 0.03 U/L for bariatric surgery). Line plots indicating the individual change in serum PRCP activity after diet or bariatric surgery are shown in Fig 5. Serum PRCP activity correlated significantly with plasma PRCP protein concentration (0.865; p<0.001) (Fig 6). The mean plasma PRCP protein concentrations (mean ± SD) before and 6 months after bariatric surgery were 18.2 ± 3.7 ng/mL and 15.7 ± 2.7 ng/mL. We also found a significant decrease (p<0.001) in the mean plasma PRCP protein concentration (mean ± SD; 2.43 ± 2.26 ng/mL) upon weight loss.

In the bariatric surgery group significant positive correlations were found between the decrease in serum PRCP activity and the decrease in body weight, BMI, total abdominal adipose tissue, visceral abdominal adipose tissue and subcutaneous abdominal adipose tissue. An inverse correlation was found with the increase in high-density lipoprotein cholesterol. Additional positive correlations with the decrease in waist and hip circumference, total cholesterol, low-density lipoprotein cholesterol and baseline glucose level were found in the diet group. These results are all summarized in Table 4.

**Table 4:** Correlations between the decrease in PRCP activity and changes in several metabolic and biochemical parameters after diet or bariatric surgery (*p ≤ 0.05, **p ≤ 0.01).

Parameter	Bariatric surgery		Diet	
	Pearson correlation	p*-*value	Pearson correlation	p*-*value
**ΔBody weight (kg)**	0.472	0.020*	0.389	0.008**
**ΔBMI (kg/m**^**2**^**)**	0.405	0.050*	0.385	0.009**
**ΔWaist (cm)**	0.293	0.164	0.332	0.026*
**ΔHip (cm)**	0.230	0.280	0.402	0.006**
**ΔVisceral fat (cm**^**2**^**)**	0.472	0.020*	0.383	0.010**
**ΔSubcutaneous fat (cm**^**2**^**)**	0.253	0.232	0.326	0.031*
**ΔTotal abdominal fat (cm**^**2**^**)**	0.414	0.044*	0.381	0.011*
**ΔTotal cholesterol (mg/dL)**	-0.148	0.490	0.420	0.004**
**ΔHDL cholesterol (mg/dL)**	-0.498	0.013*	0.174	0.253
**ΔLDL cholesterol (mg/dL)**	-0.116	0.606	0.377	0.011*
**ΔFasting glucose (mg/dL)**	0.361	0.083	0.314	0.035*

## Discussion

The presence of PRCP protein and activity in the circulation has previously been reported, but the distribution and possible *in vivo* relevance of circulating PRCP remained unclear [18,20,25,27].

The first aim of this study was to unravel the distribution of PRCP activity in human circulation. We found that white blood cells and plasma contributed the most to the circulating PRCP activity. In contrast, very low PRCP activity was found in resting and thrombin-activated blood platelets. Our next experiments were focused on finding which cells were potentially responsible for the plasma activity and which subsets of white blood cells significantly expressed PRCP. The highest PRCP activity was measured in aortic smooth muscle cells, followed by M1 macrophages, granulocytes, aortic endothelial cells and lymphocytes. A significant upregulation of PRCP activity was observed upon monocyte-to-M1-macrophage differentiation. M1 macrophages are considered to be the pro-inflammatory macrophages, which might indicate a function for PRCP in the inflammatory response [28,29]. This enzyme is thought to exert its function in inflammation via activation of the prekallikrein pathway with subsequent bradykinin formation [3,30,31]. Also, previous reports have already linked plasma PRCP to C-reactive protein and several inflammatory cytokines [20,27]. However, in our study populations no significant correlations with serum PRCP activity and C-reactive protein levels were found. Another explanation for the increased PRCP activity in macrophages could be that monocyte-to-macrophage differentiation is associated with an increase in cytoplasmic volume and more specifically with the development of larger lysosomal and mitochondrial structures to enhance their degradative capacity [32–34]. Activation of M1 macrophages through the classical LPS/IFN-γ pathway leads to a decrease in PRCP activity, indicating that PRCP could potentially be secreted. However, PRCP activity levels were undetectable in the cell culture medium. A more pronounced decrease in PRCP activity was found after degranulation of granulocytes caused by PMA-stimulation. Once more, these results point to a granular localisation of PRCP and a possible function in the immune response [18]. The highest PRCP activity was found in cytotoxic T cells compared to helper T cells and B cells. Furthermore, PRCP activity remained unchanged after TNFα stimulation of human aortic endothelial and smooth muscle cells [35–36].

The development of a sensitive immunoassay by Xu *et al*. has demonstrated that plasma protein concentrations of PRCP are increased in obese patients and are even more elevated in patients with both obesity and diabetes. Strong correlations with metabolic (BMI, blood glucose, etc.) parameters have been reported [18]. Complementary to this study, we investigated PRCP activity in the serum of normal, overweight, obese and morbidly obese patients. We found that serum PRCP activity was increased with a rising BMI the PRCP activity in serum of the groups with elevated BMI is significantly elevated compared to the PRCP activity in the lean group. In addition to correlations with BMI, body weight, waist and hip circumference, we also found strong associations with the amount of total, visceral and subcutaneous abdominal adipose tissue. The ratio of PRCP protein concentration to activity in serum could reflect the severity of obesity and sheds some much needed light on the possible additional source(s) of circulating PRCP. Moreover, this could potentially aid in the determination of PRCP’s potential role in regulating levels of intact apelin and α-MSH, two peptides involved in the regulation of food intake.

Since polymorphisms of the PRCP gene have been related to metabolic syndrome among coronary artery disease patients [37], we also categorized the patients based on metabolic syndrome. However, no significant difference in PRCP activity between the MS- and MS+ groups were found. Whether the existence of metabolic syndrome affects circulating PRCP activity specifically in patients with coronary artery disease remains to be investigated and will be addressed in future studies.

Aside from investigating PRCP activity in overweight and obese patients, we also explored its profile after weight loss. Thus, PRCP activity was measured in 24 patients who underwent bariatric surgery and 45 patients who were put on a calorie-restricted diet. PRCP activity and body weight decreased significantly after diet but even more pronounced after bariatric surgery, which is also associated with several dietary guidelines and restrictions [38]. Again, significant correlations were found with parameters concerning weight, waist and hip circumference and the amount of adipose tissue. The role of PRCP in adipocytes deserves further study, especially because adipocytes produce and secrete apelin-13, a substrate of PRCP. A strong correlation between the serum PRCP activity and plasma protein concentration was found, thus confirming the specificity of our activity measurements. Moreover, we confirmed the influence of weight loss on plasma PRCP protein concentrations. Our results suggested that both PRCP activity, as well as PRCP protein concentration, can be used to investigate the regulation of PRCP in plasma.

On the other hand, endothelial dysfunction, as observed in obese patients [39], may also lie at the basis of the raised PRCP protein concentrations in plasma, since PRCP has been shown to be located on the membrane of endothelial cells and to regulate endothelial cell growth [18,40].

## Conclusion

Our study was the first comprehensive investigation of the distribution of PRCP activity in human circulation. We have demonstrated that white blood cells and plasma contribute the most to circulating PRCP activity. Serum PRCP activity was found to correlate with plasma PRCP protein concentration, body weight and the amount of adipose tissue. These findings, taken together with the recent observation that the adipokine apelin-13 is hydrolysed by PRCP, underscore the importance of further research needed in this field to fully understand its regulation and biological function in body weight control. Gaining a greater understanding of PRCP’s multi-faceted biological functions could open up novel therapeutic avenues for the treatment of obesity.

## Supporting information

S1 Appendix PRCP activity in peripheral blood mono- and polymorphonuclear cells. (DOCX)

S2 Appendix Study populations. (DOCX)

S3 Appendix PRCP and DPP2 activity in primary murine cells. (DOCX)

S4 Appendix PRCP in a rodent model of metabolic syndrome. (DOCX)
