# Supplementary material for: Prolyl carboxypeptidase activity in the circulation and its correlation with body weight and adipose tissue in lean and obese subjects
Source: PLoS One. 2018 May 17;13(5):e0197603. doi: 10.1371/journal.pone.0197603 (PMC5957431; doi:10.1371/journal.pone.0197603)
Supplement: S1 Appendix — (DOCX) [file pone.0197603.s001.docx]

**S1. PRCP activity in peripheral blood mono- and polymorphonuclear cells.**

**1.1 Human lymphocyte isolation**

CD4^+^ T cells, CD8^+^ T cells and CD19^+^ B cells were isolated from PBMCs using Dynabeads FlowComp^TM^ Human CD4, CD8 and CD19 pan B (Life Technologies), according to the manufacturer’s instructions. For CD4^+^ and CD8^+^ T cell isolation, 0.5 x 10^7^ PBMCs were incubated for 10 min at 4 °C with human CD4 or human CD8 antibody, and thereafter were washed with 1 mL DPBS and centrifuged for 8 min at 350 *g*. The supernatant was discarded and the pellet was resuspended in 1 mL DPBS. Then, 75 μL washed FlowComp^TM^ Dynabeads were added, mixed and incubated for 15 min at room temperature under rolling and tilting. 1 mL DPBS was added and the tubes were placed in the magnet for 2 min. While the tube was still in the magnet, the supernatant containing CD4 or CD8 negative cells was removed. The bead-bound CD4^+^ or CD8^+^ T cells were lysed in enzyme activity lysis buffer. The isolation of CD19^+^ B cells is based on the same principle with 1 x 10^7^ PBMCs as starting material.

**1.2 Human granulocyte isolation and activation**

For granulocyte isolation, the third layer containing Ficoll-Paque was removed, leaving only the bottom pellet of polymorphonuclear granulocytes and red blood cells intact. This red blood cell pellet was immediately diluted in 5 mL PBS for further processing. Next, dextran sedimentation was performed by mixing the diluted red blood cell pellet with 10 mL of a 6% dextran (Sigma-Aldrich) solution in water. Optimal red blood cell sedimentation was achieved after 30 min at ambient temperature in a dark room. The upper layer, rich in granulocytes, was collected and centrifuged for 5 min at 600 *g* (no brakes). In order to obtain a pure granulocyte population, red blood cell lysis was performed by addition of 10 mL red blood cell lysis buffer to the granulocyte pellet for 20 sec. The lysis was ended by addition of 20 mL PBS and centrifugation of the suspension for 10 min at 400 *g* to obtain a well-defined pellet. According to Maqbool *et al.* > 95% of the isolated cells are neutrophils [1]. For confirmation, hematoxylin and eosin staining was performed. Next, granulocytes were activated by incubating the cells for 30 min with 10 μM phorbol 12-myristate 13-acetate (PMA, Sigma-Aldrich) at 37 °C under 5% CO_2._ Supernatants were collected and the cells were lysed for activity measurements.

**References**

1. Maqbool M, Vidyadaran S, George E, Ramasamy R. Optimisation of laboratory procedures for isolating human peripheral blood derived neutrophils. Med J Malaysia. 2011;66:296–299.
