# Supplementary material for: Prolyl carboxypeptidase activity in the circulation and its correlation with body weight and adipose tissue in lean and obese subjects
Source: PLoS One. 2018 May 17;13(5):e0197603. doi: 10.1371/journal.pone.0197603 (PMC5957431; doi:10.1371/journal.pone.0197603)
Supplement: S2 Appendix — (DOCX) [file pone.0197603.s002.docx]

**S2. Study populations.**

**2.1 Individual data points**

**Table 2.1 Individual data points of study cohort 1**

| **Patient** | **Age (years)** | **Weight (kg)** | **Height (m)** | **BMI (kg/m²)** | **PRCP activity (U/L)** |
| --- | --- | --- | --- | --- | --- |
| 1 | 47 | 52,4 | 1,535 | 22,2 | 0,98 |
| 2 | 32 | 63,0 | 1,715 | 21,4 | 0,7 |
| 3 | 38 | 60,8 | 1,670 | 21,8 | 0,86 |
| 4 | 30 | 53,4 | 1,645 | 19,7 | 0,74 |
| 5 | 43 | 57,2 | 1,730 | 19,1 | 0,9 |
| 6 | 19 | 60,2 | 1,720 | 20,3 | 0,84 |
| 7 | 26 | 59,6 | 1,585 | 23,7 | 0,75 |
| 8 | 42 | 57,0 | 1,615 | 21,9 | 1,08 |
| 9 | 26 | 52,6 | 1,655 | 19,2 | 0,82 |
| 10 | 50 | 68,0 | 1,730 | 22,7 | 0,92 |
| 11 | 42 | 69,2 | 1,710 | 23,7 | 0,93 |
| 12 | 47 | 58,4 | 1,605 | 22,7 | 0,52 |
| 13 | 45 | 56,2 | 1,645 | 20,8 | 0,53 |
| 14 | 46 | 58,8 | 1,620 | 22,4 | 1,10 |
| 15 | 26 | 58,6 | 1,700 | 20,3 | 0,84 |
| 16 | 52 | 77,0 | 1,610 | 29,7 | 1,17 |
| 17 | 69 | 63,8 | 1,540 | 26,9 | 0,88 |
| 18 | 58 | 71,6 | 1,600 | 28,0 | 1,45 |
| 19 | 51 | 123,6 | 1,575 | 49,8 | 1,22 |
| 20 | 47 | 103,6 | 1,655 | 37,8 | 1,26 |
| 21 | 46 | 79,0 | 1,675 | 28,2 | 0,88 |
| 22 | 24 | 83,4 | 1,730 | 27,9 | 1,02 |
| 23 | 18 | 82,8 | 1,665 | 29,9 | 0,86 |
| 24 | 34 | 113,8 | 1,670 | 40,8 | 0,95 |
| 25 | 38 | 101,8 | 1,590 | 40,3 | 1,01 |
| 26 | 47 | 85,6 | 1,675 | 30,5 | 0,87 |
| 27 | 37 | 91,8 | 1,600 | 35,9 | 0,92 |
| 28 | 23 | 115,0 | 1,760 | 37,1 | 0,94 |
| 29 | 20 | 68,8 | 1,595 | 27,0 | 1,06 |
| 30 | 51 | 75,2 | 1,595 | 29,6 | 1,07 |
| 31 | 40 | 94,2 | 1,630 | 35,5 | 1,14 |
| 32 | 57 | 85,0 | 1,580 | 34,0 | 1,19 |
| 33 | 34 | 104,4 | 1,665 | 37,7 | 1,26 |
| 34 | 26 | 100,0 | 1,650 | 36,7 | 1,19 |
| 35 | 43 | 82,4 | 1,675 | 29,4 | 0,99 |
| 36 | 20 | 76,6 | 1,715 | 26,0 | 0,72 |
| 37 | 68 | 121,0 | 1,615 | 46,4 | 1,29 |
| 38 | 30 | 85,4 | 1,700 | 29,6 | 1,19 |
| 39 | 41 | 86,8 | 1,760 | 28,0 | 1,16 |
| 40 | 59 | 87,2 | 1,615 | 33,4 | 1,15 |
| 41 | 29 | 70,0 | 1,625 | 26,5 | 1,15 |
| 42 | 33 | 88,2 | 1,650 | 32,4 | 1,03 |
| 43 | 37 | 110,8 | 1,660 | 40,2 | 1,09 |
| 44 | 53 | 73,2 | 1,670 | 26,2 | 1,33 |
| 45 | 55 | 78,2 | 1,710 | 26,7 | 1,15 |
| 46 | 56 | 79,8 | 1,680 | 28,3 | 1,04 |
| 47 | 57 | 84,4 | 1,550 | 35,1 | 1,03 |
| 48 | 38 | 122,4 | 1,665 | 44,2 | 1,22 |
| 49 | 41 | 96,4 | 1,530 | 41,2 | 1,39 |
| 50 | 20 | 140,8 | 1,650 | 51,4 | 1,12 |
| 51 | 19 | 100,8 | 1,560 | 41,2 | 1,00 |
| 52 | 64 | 99,2 | 1,705 | 34,1 | 1,26 |
| 53 | 65 | 126,0 | 1,600 | 49,2 | 0,98 |
| 54 | 55 | 73,4 | 1,610 | 28,3 | 1,21 |
| 55 | 49 | 98,0 | 1,710 | 33,5 | 1,02 |
| 56 | 46 | 90,6 | 1,635 | 33,9 | 1,38 |
| 57 | 54 | 123,8 | 1,670 | 44,4 | 0,84 |
| 58 | 48 | 83,4 | 1,655 | 30,4 | 1,18 |
| 59 | 45 | 94,8 | 1,720 | 32,0 | 1,26 |
| 60 | 54 | 126,0 | 1,675 | 44,9 | 1,07 |
| 61 | 41 | 89,4 | 1,755 | 29,0 | 0,84 |
| 62 | 43 | 78,8 | 1,675 | 28,1 | 0,74 |
| 63 | 21 | 130,4 | 1,630 | 49,1 | 1,65 |
| 64 | 56 | 121,8 | 1,705 | 41,9 | 1,38 |
| 65 | 39 | 116,4 | 1,630 | 43,8 | 1,40 |
| 66 | 18 | 129,8 | 1,875 | 36,9 | 1,38 |
| 67 | 50 | 93,6 | 1,600 | 36,6 | 1,16 |
| 68 | 41 | 128,2 | 1,650 | 47,1 | 1,47 |
| 69 | 40 | 127,2 | 1,685 | 44,8 | 1,22 |
| 70 | 25 | 112,0 | 1,735 | 37,2 | 0,98 |
| 71 | 21 | 120,8 | 1,725 | 40,6 | 1,48 |
| 72 | 24 | 105,2 | 1,575 | 42,4 | 1,25 |
| 73 | 52 | 104,4 | 1,665 | 37,7 | 1,11 |
| 74 | 47 | 73,8 | 1,540 | 27,0 | 0,88 |
| 75 | 31 | 138,8 | 1,780 | 43,6 | 1,66 |

**Table 2.2 Individual data points of study cohort 2**

| **Patient** | **Treatment** | **Height**  **(m)** | **Age (years)** | **Post**  **Weight**  **(kg)** | **Pre**  **Weight (kg)** | **Delta**  **Weight**  **(kg)** | **Post**  **BMI (kg/m²)** | **Pre**  **BMI (kg/m²)** | **Delta**  **BMI (kg/m²)** | **Pre**  **PRCP activity (U/L)** | **Post**  **PRCP activity (U/L)** | **Delta**  **PRCP activity (U/L)** |
| --- | --- | --- | --- | --- | --- | --- | --- | --- | --- | --- | --- | --- |
| 1 | Diet | 1,67 | 24 | 84 | 97 | 13 | 30,1 | 34,8 | 4,7 | 0,74 | 1,01 | 0,27 |
| 2 | Diet | 1,63 | 46 | 76,8 | 104,2 | 27,4 | 28,9 | 39,2 | 10,3 | 1,05 | 1,29 | 0,24 |
| 3 | Diet | 1,61 | 38 | 68 | 82 | 14 | 26,2 | 31,6 | 5,4 | 0,72 | 0,8 | 0,08 |
| 4 | Diet | 1,62 | 20 | 73 | 78 | 5 | 27,8 | 29,7 | 1,9 | 0,98 | 0,94 | -0,04 |
| 5 | Diet | 1,665 | 62 | 81,8 | 84,2 | 2,4 | 29,5 | 30,4 | 0,9 | 1,19 | 0,86 | -0,33 |
| 6 | Diet | 1,675 | 40 | 85,6 | 100,6 | 15 | 30,5 | 35,9 | 5,4 | 0,67 | 0,57 | -0,1 |
| 7 | Bariatric surgery | 1,6 | 26 | 68,8 | 90 | 21,2 | 26,9 | 35,2 | 8,3 | 0,79 | 0,82 | 0,03 |
| 8 | Bariatric surgery | 1,615 | 38 | 95,2 | 105,4 | 10,2 | 36,5 | 40,4 | 3,9 | 1,11 | 1,16 | 0,05 |
| 9 | Bariatric surgery | 1,565 | 30 | 97,4 | 120 | 22,6 | 39,8 | 49 | 9,2 | 1,09 | 1,49 | 0,4 |
| 10 | Diet | 1,715 | 63 | 100 | 119 | 19 | 34 | 40,5 | 6,5 | 0,76 | 0,83 | 0,07 |
| 11 | Diet | 1,62 | 43 | 75,8 | 91,4 | 15,6 | 28,9 | 34,8 | 5,9 | 0,67 | 1,02 | 0,35 |
| 12 | Bariatric surgery | 1,66 | 45 | 85,8 | 109,8 | 24 | 31,1 | 39,8 | 8,7 | 1,06 | 1,28 | 0,22 |
| 13 | Diet | 1,69 | 50 | 80,4 | 82,6 | 2,2 | 28,2 | 28,9 | 0,7 | 0,67 | 0,75 | 0,08 |
| 14 | Diet | 1,605 | 55 | 64,4 | 68,8 | 4,4 | 25 | 26,7 | 1,7 | 0,78 | 0,78 | 0 |
| 15 | Diet | 1,64 | 46 | 78,8 | 80 | 1,2 | 29,3 | 29,7 | 0,4 | 0,77 | 0,71 | -0,06 |
| 16 | Bariatric surgery | 1,63 | 31 | 76,6 | 102,8 | 26,2 | 28,8 | 38,7 | 9,9 | 0,9 | 0,83 | -0,07 |
| 17 | Diet | 1,73 | 45 | 80 | 89,6 | 9,6 | 26,7 | 29,8 | 3,1 | 1 | 1,02 | 0,02 |
| 18 | Bariatric surgery | 1,645 | 20 | 115,6 | 158,4 | 42,8 | 42,7 | 58,5 | 15,8 | 0,98 | 1,17 | 0,19 |
| 19 | Bariatric surgery | 1,7 | 19 | 118,6 | 150,2 | 31,6 | 41 | 52 | 11 | 0,89 | 1,14 | 0,25 |
| 20 | Diet | 1,58 | 67 | 73,2 | 74,8 | 1,6 | 29,3 | 30 | 0,7 | 1 | 0,95 | -0,05 |
| 21 | Bariatric surgery | 1,66 | 31 | 99,2 | 134,8 | 35,6 | 36 | 48,9 | 12,9 | 1,25 | 1,53 | 0,28 |
| 22 | Bariatric surgery | 1,695 | 35 | 91,8 | 120,2 | 28,4 | 32 | 41,8 | 9,8 | 0,76 | 1,25 | 0,49 |
| 23 | Diet | 1,64 | 53 | 107,2 | 119,4 | 12,2 | 39,9 | 44,4 | 4,5 | 0,98 | 1,29 | 0,31 |
| 24 | Bariatric surgery | 1,675 | 53 | 81 | 109,8 | 28,8 | 28,9 | 39,1 | 10,2 | 1,08 | 1,49 | 0,41 |
| 25 | Bariatric surgery | 1,69 | 42 | 83,8 | 105,8 | 22 | 29,3 | 37 | 7,7 | 0,86 | 1,12 | 0,26 |
| 26 | Diet | 1,585 | 52 | 78,8 | 84,8 | 6 | 31,4 | 33,8 | 2,4 | 0,91 | 0,94 | 0,03 |
| 27 | Diet | 1,61 | 42 | 71,6 | 74,8 | 3,2 | 27,6 | 28,9 | 1,3 | 0,98 | 0,94 | -0,04 |
| 28 | Bariatric surgery | 1,665 | 57 | 81,4 | 107,4 | 26 | 29,4 | 38,5 | 9,1 | 0,88 | 1,01 | 0,13 |
| 29 | Diet | 1,665 | 39 | 72,6 | 91,2 | 18,6 | 26,2 | 32,9 | 6,7 | 0,82 | 1,13 | 0,31 |
| 30 | Bariatric surgery | 1,725 | 43 | 110,8 | 138,4 | 27,6 | 37,2 | 46,5 | 9,3 | 0,68 | 0,85 | 0,17 |
| 31 | Diet | 1,53 | 26 | 82 | 91,2 | 9,2 | 35 | 39 | 4 | 1,11 | 1,04 | -0,07 |
| 32 | Bariatric surgery | 1,6 | 45 | 82,2 | 100,8 | 18,6 | 32,1 | 39,4 | 7,3 | 0,65 | 0,63 | -0,02 |
| 33 | Diet | 1,705 | 67 | 90,2 | 97,8 | 7,6 | 31 | 33,3 | 2,3 | 0,97 | 1,27 | 0,3 |
| 34 | Diet | 1,59 | 57 | 89,8 | 112,2 | 22,4 | 35,5 | 44,4 | 8,9 | 0,63 | 0,58 | -0,05 |
| 35 | Diet | 1,59 | 51 | 89,4 | 89,8 | 0,4 | 35,4 | 35,2 | -0,2 | 1,44 | 1,29 | -0,15 |
| 36 | Bariatric surgery | 1,65 | 64 | 92,8 | 112,6 | 19,8 | 34,1 | 41,4 | 7,3 | 0,87 | 1,01 | 0,14 |
| 37 | Diet | 1,63 | 70 | 89 | 99,4 | 10,4 | 33,5 | 36,8 | 3,3 | 1,18 | 1,18 | 0 |
| 38 | Diet | 1,58 | 58 | 68,8 | 76,8 | 8 | 27,6 | 30,8 | 3,2 | 1,14 | 1,97 | 0,83 |
| 39 | Diet | 1,58 | 59 | 78 | 90,2 | 12,2 | 31,2 | 36,1 | 4,9 | 0,84 | 1,12 | 0,28 |
| 40 | Diet | 1,66 | 38 | 61,4 | 85,6 | 24,2 | 22,3 | 31,1 | 8,8 | 0,93 | 1,22 | 0,29 |
| 41 | Bariatric surgery | 1,56 | 24 | 67,6 | 110,6 | 43 | 27,8 | 45,4 | 17,6 | 0,69 | 1,08 | 0,39 |
| 42 | Diet | 1,69 | 56 | 82,8 | 102 | 19,2 | 29 | 35,7 | 6,7 | 0,6 | 0,92 | 0,32 |
| 43 | Diet | 1,61 | 62 | 86,6 | 92,6 | 6 | 33,4 | 35,7 | 2,3 | 0,82 | 0,95 | 0,13 |
| 44 | Diet | 1,64 | 63 | 84 | 95,6 | 11,6 | 31,2 | 35,5 | 4,3 | 0,94 | 0,94 | 0 |
| 45 | Diet | 1,68 | 35 | 127,4 | 131,2 | 3,8 | 45,1 | 46,5 | 1,4 | 1,06 | 1,25 | 0,19 |
| 46 | Diet | 1,675 | 36 | 87 | 99,8 | 12,8 | 31 | 35,6 | 4,6 | 0,79 | 0,75 | -0,04 |
| 47 | Bariatric surgery | 1,585 | 25 | 74 | 94,6 | 20,6 | 29,5 | 37,7 | 8,2 | 0,68 | 0,7 | 0,02 |
| 48 | Diet | 1,58 | 31 | 80,8 | 93 | 12,2 | 32,4 | 37,3 | 4,9 | 0,77 | 0,82 | 0,05 |
| 49 | Diet | 1,59 | 57 | 86 | 96,6 | 10,6 | 34 | 38,2 | 4,2 | 0,88 | 0,94 | 0,06 |
| 50 | Diet | 1,65 | 46 | 79,6 | 92,2 | 12,6 | 29,2 | 33,9 | 4,7 | 0,71 | 1,13 | 0,42 |
| 51 | Bariatric surgery | 1,65 | 54 | 99,2 | 133,4 | 34,2 | 36,4 | 49 | 12,6 | 0,94 | 1,24 | 0,3 |
| 52 | Diet | 1,745 | 37 | 140 | 138,1 | -1,9 | 46 | 45,4 | -0,6 | 0,87 | 0,71 | -0,16 |
| 53 | Bariatric surgery | 1,655 | 32 | 68,8 | 92,8 | 24 | 25,1 | 33,5 | 8,4 | 1,06 | 1,4 | 0,34 |
| 54 | Diet | 1,61 | 48 | 81,6 | 84 | 2,4 | 31,5 | 32,4 | 0,9 | 1,04 | 0,88 | -0,16 |
| 55 | Diet | 1,7 | 26 | 108 | 115,2 | 7,2 | 37,4 | 39,9 | 2,5 | 1,17 | 0,91 | -0,26 |
| 56 | Diet | 1,73 | 35 | 102 | 121 | 19 | 34,1 | 40,4 | 6,3 | 1,25 | 1,26 | 0,01 |
| 57 | Diet | 1,64 | 49 | 106,8 | 114,8 | 8 | 39,7 | 42,7 | 3 | 1,17 | 1,19 | 0,02 |
| 58 | Diet | 1,85 | 43 | 128,2 | 138,2 | 10 | 37,5 | 40,4 | 2,9 | 0,94 | 1,19 | 0,25 |
| 59 | Diet | 1,61 | 38 | 75,8 | 97,8 | 22 | 29,2 | 37,7 | 8,5 | 0,71 | 0,7 | -0,01 |
| 60 | Diet | 1,67 | 22 | 83,2 | 85,4 | 2,2 | 29,8 | 30,6 | 0,8 | 1,07 | 0,9 | -0,17 |
| 61 | Bariatric surgery | 1,56 | 46 | 78,2 | 100,4 | 22,2 | 32,1 | 41,3 | 9,2 | 0,9 | 0,96 | 0,06 |
| 62 | Bariatric surgery | 1,54 | 55 | 100,4 | 120,6 | 20,2 | 42,3 | 50,9 | 8,6 | 1,07 | 1,01 | -0,06 |
| 63 | Diet | 1,735 | 56 | 94,2 | 105,4 | 11,2 | 31,3 | 35 | 3,7 | 0,77 | 0,78 | 0,01 |
| 64 | Diet | 1,615 | 33 | 81 | 97,4 | 16,4 | 31,1 | 37,3 | 6,2 | 0,72 | 0,97 | 0,25 |
| 65 | Diet | 1,67 | 44 | 75,4 | 80,8 | 5,4 | 27 | 29 | 2 | 0,89 | 0,91 | 0,02 |
| 66 | Bariatric surgery | 1,655 | 26 | 71,6 | 104,2 | 32,6 | 26,1 | 38 | 11,9 | 0,93 | 1,15 | 0,22 |
| 67 | Diet | 1,68 | 43 | 86,8 | 83,4 | -3,4 | 30,8 | 29,5 | -1,3 | 0,8 | 0,82 | 0,02 |
| 68 | Bariatric surgery | 1,725 | 41 | 103,6 | 121,6 | 18 | 34,8 | 40,9 | 6,1 | 0,8 | 0,95 | 0,15 |
| 69 | Bariatric surgery | 1,675 | 47 | 71 | 106 | 35 | 25,3 | 37,8 | 12,5 | 0,74 | 0,89 | 0,15 |

**2.2 Inclusion criteria and metabolic work-up**

All patients were female and predominantly of Western European descent. At enrolment none were involved in a weight management program. Subjects had to be 18 years or older. Both pre- and postmenopausal women were included in the analyses. Menopause was defined using clinical data (no menstruation during the previous year) combined with hormonal data (FSH > 25 mU/mL and estradiol < 20 pg/mL). Patients were defined as former smokers if they stopped smoking more than one year prior to the metabolic work-up. As diabetes has a specific clinical feature and longstanding treatments are potential confounders, patients already known to have diabetes were not included. Glucose tolerance status was defined based on the criteria of the American Diabetes Association (2013) [1].

The metabolic work-up included a detailed questionnaire and a clinical examination with anthropometry. All anthropometric measurements were performed in the morning, with patients in fasting conditions and undressed. Height was measured to the nearest 0.5 cm and body weight was measured with a digital scale to the nearest 0.2 kg. BMI was calculated as weight (in kilograms) over height (in meter) squared. Waist circumference was measured at the mid-level between the lower rib margin and the iliac crest. Hip circumference was measured at the level of the trochanter major. Waist-hip ratio was calculated by dividing waist circumference by hip circumference. The cross-sectional areas of total abdominal adipose tissue (TAT), visceral abdominal adipose tissue (VAT) and subcutaneous abdominal adipose tissue (SAT) were measured by CT at L4-L5 level according to previously described methods [2]. First, the total area of abdominal adipose tissue was measured at -190 to -30 Hounsfield Units. Subsequently, the area of VAT was distinguished from SAT by manually tracking the abdominal muscular wall separating the two adipose tissue compartments. The area of VAT was measured and the area of SAT was calculated by subtracting the area of VAT from the total area of TAT. Systolic and diastolic blood pressure was determined on the right arm of the patient, after at least 5 min rest, using a mercury sphygmomanometer. A fasting blood analysis (taken from an antecubital vein) included high-sensitive C-reactive protein (hs-CRP), glycated haemoglobin A1 (HbA1c) and lipid profile [total cholesterol and high-density lipoprotein cholesterol (HDL-C), and triglycerides. LDL-C was calculated using the Friedewald formula [3]. A 3-h oral glucose tolerance test with 75 g of glucose with sampling at 0, 15, 30, 60, 90, 120, 150 and 180 minutes was carried out, insulin was also determined at 0, 30, 60, 120 and 180 minutes. Plasma glucose, total cholesterol and TG were measured on Vitros 750 XRC (Ortho Clinical Diagnostics, Johnson & Johnson). HDL-C was measured on Hitachi 912 (Roche Diagnostics). Insulin levels were measured with the Medgenic two-site IRMA assay (BioSource). HbA1c was determined by high performance liquid chromatography (Adams™ A1c HA- 8180, Arkray–Menarini instrument; reference range: 4.8-6.0%). Hs-CRP was assayed with nephelometry on BNII (Siemens Healthcare Diagnostics).

Following their baseline assessment, patients entered a follow-up weight loss program. Patients considered for bariatric surgery (some of them on their own request) and meeting the Belgian eligibility criteria for bariatric surgery (BMI > 35 kg/m² with co-morbidities (diabetes mellitus or obstructive sleep apnea or therapy resistant arterial hypertension) or BMI > 40 kg/m² with or without co-morbidities) underwent an extensive preoperative multidisciplinary evaluation according to Belgian recommendations (KB 2010). The type of surgery was discussed with the patient after a comprehensive multidisciplinary evaluation including information about the risks and benefits of the various options. Patients underwent an adjustable gastric banding procedure (a strictly restrictive procedure, n=1) or a roux-en-Y gastric bypass procedure with an alimentary limb of 100-120 cm (a more complex intervention that is associated with restriction, malabsorption, and gut hormone modulation, n=23). After bariatric surgery, patients were advised to follow a balanced low-calorie diet. Patients who did not undergo bariatric surgery entered a weight management program. In this protocol, overweight and obese patients followed a kitchen-prepared very low calorie diet (VLCD) or a hypocaloric diet. The first 6 weeks, the VLCD had an energy content of approximately 650 kcal with 45 grams of carbohydrates daily. In a second phase of 6 to 12 weeks, the carbohydrate content of the diet was increased to 82 grams. In a third phase, after 3 to 6 months, the energy content of the diet was determined on an individual basis, with a lower limit of 1200 kcal. The hypocaloric diet for the non-VLCD group was calculated as resting metabolic rate x physical activity level - 600 kcal. Resting metabolic rate was measured using indirect calorimetry or estimated using the Harris-Benedict equation [4]. The physical activity level was defined to be 1.3 or 1.4, based on the physical activity of the individual patient. A skilled dietician gave dietary advice and patients were stimulated to be physically active.

**References**

1. American Diabetes Association. Diagnosis and classification of diabetes mellitus. Diabetes Care. 2010;33:62-69.

2. Van der Kooy K, Seidell J. Techniques for the measurement of visceral fat: a practical guide. Int J Obes Relat Metab Disord. 1993;17:187–196.

3. Friedewald WT, Levy RI, Fredrickson DS. Estimation of the concentration of low-density lipoprotein cholesterol in plasma, without use of the preparative ultracentrifuge. Clin Chem. 1972;18:499–502.

4. Harris JA, Benedict FG. A biometric study of human basal metabolism. Proc Natl Acad Sci. 1918;4:370–373.
