# Supplementary material for: Prolyl carboxypeptidase activity in the circulation and its correlation with body weight and adipose tissue in lean and obese subjects
Source: PLoS One. 2018 May 17;13(5):e0197603. doi: 10.1371/journal.pone.0197603 (PMC5957431; doi:10.1371/journal.pone.0197603)
Supplement: S3 Appendix — (DOCX) [file pone.0197603.s003.docx]

**S3. PRCP and DPP2 activity in primary murine cells.**

**3.1 Murine bone marrow-derived monocyte and macrophage isolation**

Monocytes and macrophages were derived from the bone marrow of Swiss mice (Jackson Laboratories). After sacrifice, the tibias and femurs of the mice were isolated and immersed in DPBS. The end of each bone was cut off and the bone marrow was flushed from both ends of the bones with RPMI-1640 supplemented with 10% heat-inactivated FCS, 100 U/mL penicilin, 100 μg/mL streptomycin (Life Technologies) and 10 U/mL heparin (Sigma-Aldrich). Cells were passed through a 40 μM cell strainer and subsequently washed twice in HEPES-buffered RPMI 1640. Cells were seeded at 500 000 cells/well in 12-well plates and grown in RPMI 1640 supplemented with 10% heat-inactivated FCS, 15% L-cell conditioned medium, 100 U/mL penicilin and 100 μg/mL streptomycin at 37 °C under 5% CO_2_. Non-tissue coated plates were used for the growth of monocytes and tissue coated plates (Greiner Bio-One) for the differentation into macrophages. Medium was replaced every four days. Monocytes, the cells which grow in suspension, were harvested on day 5. The adherent cells, also called resting macrophages (M0), were detached by scraping and harvested on day 6. Further differentiation into activated macrophages (M1) was performed by stimulation of M0 macrophages with 100 ng/mL LPS and 100 U/mL IFN-γ for 24 h at 37 °C under 5% CO_2_. Supernatants were collected and all cells were lysed for PRCP/DPP2 activity measurements. This protocol was adapted from Francke *et al* [1]*.* To verify activation of the resting macrophages, nitrite concentrations were determined in the supernatant of M1 macrophages as a measure of NO secretion. A modified Griess assay was used as described in [2,3]. A 45-μL aliquot of supernatant was first added to 40 μL of water in a 96-well plate to which then 5 μL of sulfanilic acid (0.6% in 5% phosphorous acid) was added. This was incubated for 15 min at ambient temperature on a plate shaker, after which 5 μL 0.06 % N-(1- naphthyl)ethylenediamine dihydrochloride was added and incubated for 15 min at room temperature. Absorbance was then measured at 548 nm in an InfiniteTM 200 reader (Tecan Benelux).

**3.2 Dipeptidyl peptidase 2 activity measurement**

Dipeptidyl peptidase 2 (DPP2) enzymatic activity was determined according to Maes *et al* [4]. A 10 μL sample was incubated in a 96-well plate with 190 μL of a 2 mM Lys-Ala-pNA (Bachem) solution in 0.05 cacodylic acid-NaOH buffer pH 5.5 containing 10 mM EDTA and 14 μg/mL aprotinin. DPP2 activity was determined kinetically during 10 minutes at 37 °C by measuring the initial velocites of pNA release (405 nm) from the chromogenic substrate using a Spectramax Plus Microtiterplate Reader (Molecular devices). The molar absorptivity of pNA at 405 nm is 10.2 mM^-1^cm^-1^ and the pathlength of a volume of 200 μL per 96-well is 0.5761 cm.

**3.3 PRCP western blot**

Cell lysates were further diluted in Laemmli sample buffer (4x) before loading onto a 10% SDS-PAGE gel to allow protein separation. Thereafter, the proteins were transferred to a nitrocellulose (0.45 μm) membrane by electroblotting in 25 mM Tris buffer pH 8.3 with 0.192 M glycine and 20% methanol in the Mini Trans-blot Cell Assembly (Bio-Rad). Blocking of non-specific binding sites was achieved by placing the membrane in 5% skimmed milk in washing buffer (0.05 M Tris, 0.15 M NaCl, 0.01% Tween 20) for 1 h at room temperature. The blots were then incubated overnight at 4 °C with primary antibody against PRCP (bs-1873R, 1:500 dilution, Gentaur), and vinculin (ab129002, 1:20000 dilution, Abcam), which was used as loading control. Next, the membranes were incubated with HRP-goat anti-rabbit antibodies (656120, 1:5000 dilution, Invitrogen) for 2 h at ambient temperature. Blots were washed six times for 5 min between each incubation in washing buffer. Chemiluminesecent detection was performed using the SuperSignal West Femto substrate kit from Thermo Fisher Scientific (Erembodegem). The protein bands were visualized via an OptiGo viewer and Proxima AQ-4 software (Isogen Life Sciences). Quantification of the images was done using the TotalLab software (Isogen Life Sciences).

**3.4 Statistical Analysis**

Differences in PRCP and DPP2 activity and protein expression between various cell types were analysed with Kruskal-Wallis and Mann-Whitney U-tests. P-values were corrected for multiple testing based on scientific relevant pairwise comparisons.

**3.5 Results**

Monocytes were isolated from the bone marrow of wild-type mice and differentiated into resting and activated macrophages. Both the results of enzyme activity and western blot revealed an upregulation of PRCP activity and protein expression upon monocyte-to-macrophage differentiation, though not significantly (Kruskal-Wallis, n=4). The activity of another lysosomal enzyme named DPP2 was also increased upon monocyte-to-macrophage differentiation and activation, indicating that the mechanism behind the upregulation of PRCP might not be specific. The adapted Griess assay showed significantly higher nitrite concentrations in the supernatant of M1 macrophages, indicating that the activation of M0 macrophages was successful (p = 0.029; Mann-Whitney U test, n=4) (**Figure 3.1**).

**A** **B**

**
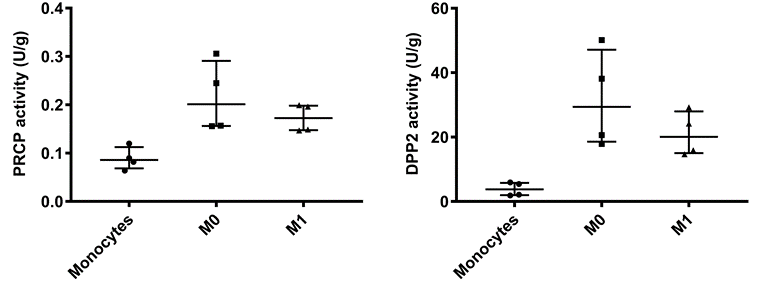
**

**
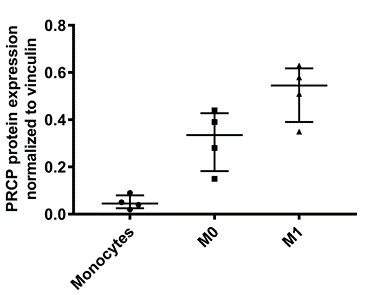
C D**

**M1**

**M0**

**Monocytes**

**
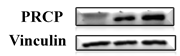
**

**E**

**
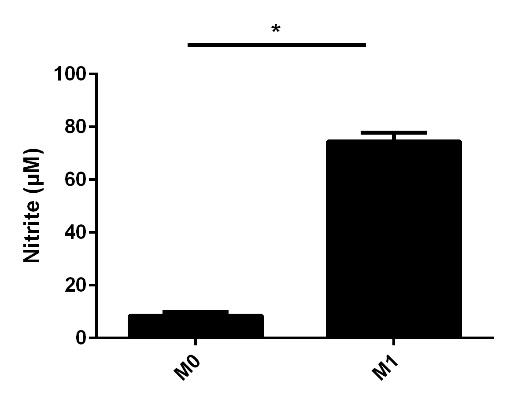
**

Figure 3.1 (A) PRCP and (B) DPP2 activity (U/g), (C, D) PRCP protein expression in murine bone marrow-derived monocytes and macrophages and Griess assay data of nitrite concentrations in the supernatant of macrophages.
(median with interquartile range, n=4)

**References**

1. Francke A, Herold J, Weinert S, Strasser RH, Braun-Dullaeus RC. Generation of mature murine monocytes from heterogeneous bone marrow and description of their properties. J Histochem Cytochem. 2011;59:813–825.

2. Waumans Y, Vliegen G, Maes L, et al. The dipeptidyl peptidases 4, 8, and 9 in mouse monocytes and macrophages: DPP8/9 Inhibition attenuates M1 macrophage activation in mice. Inflammation. 2015;39,413-424.

3. Bryan NS, Grisham MB. Methods to detect nitric oxide and its metabolites in biological samples. Free Radic Biol Med. 2007;43:645–657.

4. Maes M, Lambeir A, Gilany K, et al. Kinetic investigation of human dipeptidyl peptidase II (DPPII)-mediated hydrolysis of dipeptide derivatives and its identification as quiescent cell proline dipeptidase (QPP)/dipeptidyl peptidase 7 (DPP7). J Biochem. 2005;324:315–324
