# Supplementary material for: Prolyl carboxypeptidase activity in the circulation and its correlation with body weight and adipose tissue in lean and obese subjects
Source: PLoS One. 2018 May 17;13(5):e0197603. doi: 10.1371/journal.pone.0197603 (PMC5957431; doi:10.1371/journal.pone.0197603)
Supplement: S4 Appendix — (DOCX) [file pone.0197603.s004.docx]

**S4. PRCP in a rodent model of metabolic syndrome.**

**4.1 Animals**

International principles of laboratory animal care were followed and the experiments were approved by the local ethics committee of the University of Antwerp (study number 2013/49). 24 Wistar rats (Charles River) were kept in standard cages with an inverted 12-hour day/night cycle and were randomly divided into 3 groups (n=8 per group) fed 3 different diets **(Table 4.1)**. Rats were given *ad libitum* access to food and water. The high-fat (HF) and high-fat high-fructose (HFHF) group developed metabolic syndrome.

| **Group** | **Diet** |
| --- | --- |
| Control | Standard rodent starch diet R/M-H v1534, 9% energy from fat, 58% from carbohydrates and 33% from proteins, 3.06 kcal/g (Sniff^®^_,_ Germany) |
| High-fat | 62% energy from fat, 20% from carbohydrates and 18% from proteins, 5.10 kcal/g (TestDiet^®^, St. Louis, USA) |
| High-fat high -fructose | 46% energy from fat, 36% from carbohydrates (34% from fructose) and 18% from proteins, 4.64 kcal/g (TestDiet^®^, St. Louis, USA). The drinking water was supplemented with 25% (w:v) fructose. |

**Table 4.1** Composition of normal and hypercaloric diets.

The animals were sacrificed at 20 weeks of age using CO_2_ and peripheral tissues (kidney, subcutaneous adipose tissue) were collected and snap frozen in liquid nitrogen and kept at -80 °C. Blood samples (1.5 mL) were taken from the lateral tail vain (Multivette 600Z) after a fasting period of 12h. Blood samples were centrifuged (10000 g, 10 min) and serum was stored at -20 °C until analysis. Upon analysis, tissue samples were first cut into small pieces and homogenized (Polytron 1200E) on ice in a lysis buffer (1% octylglucoside, 10 mM EDTA, 70 μg/mL aprotinin in a 50 mM Tris buffer, pH 8.3) to measure PRCP activity. PRCP activity was measured via a RP-HPLC assay. Body weight was monitored and the plasma lipid profile was determined using diagnostic kits for the measurement of total cholesterol, HDL-cholesterol, triglycerides and plasma free fatty acid levels. Blood glucose levels were measured by a glucose analyzer and insulin by an enzyme immunoassay.

**4.2 Statistical analysis**

Differences between controls and metabolic syndrome rats were assessed by Kruskall-Wallis and Mann-Whitney U-tests. P-values were corrected for multiple testing based on scientifically relevant pairwise comparisons. Bivariate (Pearson) correlations were performed to assess the relation between serum PRCP activity and several metabolic parameters. Data are presented as median with interquartile range.

**4.3 Results**

Serum PRCP activity was not significantly different between the control, HF and HFHF group (p = 0.67). Specific PRCP activity (U/g) was determined in kidney and subcutaneous adipose tissue collected from the three different groups. The PRCP activity in subcutaneous adipose tissue was found to be significantly higher in the HF group compared to the control (p = 0.005) and HFHF group (p = 0.015). In addition, PRCP activity was found to be significantly higher in the kidney of the control (p < 0.001) and the HF (p < 0.001) group compared to the HFHF group. Results are shown in **Figure 4.1**.


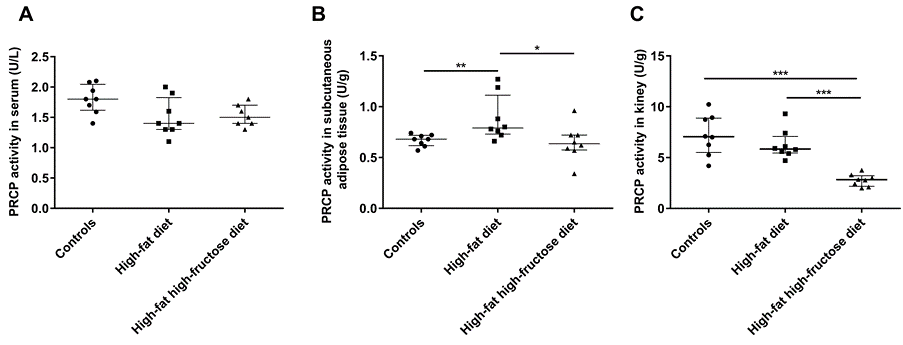


Figure 4.1 PRCP activity was measured in (A) serum, (B) subcutaneous adipose tissue and (C) kidney homogenates collected from control rats and rats fed a HF- or HFHF-diet.
(median with interquartile range, n=8 per group, *p < 0.05, **p < 0.01, ***p < 0.001)

As expected, body weight (p = 0.001), blood glucose (p = 0.018) and insulin levels (p < 0.001) were significantly higher in the HF and HFHF groups compared to the controls. No significant changes between the groups were found for total cholesterol (p = 0.116) and HDL-cholesterol (p = 0.960). The triglyceride levels were significantly higher in the HFHF group compared to the HF group (p = 0.003).

**Table 4.2** Correlations between serum PRCP activity and several metabolic parameters in rats with metabolic syndrome (n=16, **p ≤ 0.01).

| **Parameter** | **Pearson correlation** | **p-value** |
| --- | --- | --- |
| **Body weight (g)** | -0.705 | 0.003** |
| **Blood glucose (mg/dL)** | -0.290 | 0.294 |
| **Insulin (pmol/L)** | 0.208 | 0.440 |
| **Total Cholesterol (mg/dL)** | 0.393 | 0.147 |
| **HDL-cholesterol (mg/dL)** | 0.362 | 0.185 |
| **Triglycerides (mg/dL)** | -0.137 | 0.626 |
| **PRCP_adipose_ (U/g)** | -0.031 | 0.904 |
| **PRCP_kidney_ (U/g)** | 0.033 | 0.906 |
|  | | |

The correlations of serum PRCP activity with several metabolic parameters in rats with metabolic syndrome are summarized in the **Table 4.2**. In contrast to the results in human patients, a significant inverse correlation for serum PRCP activity and body weight was found **(Figure 4.2)**. This may indicate species differences between rodents and humans in the regulation of PRCP and warrants further investigation.


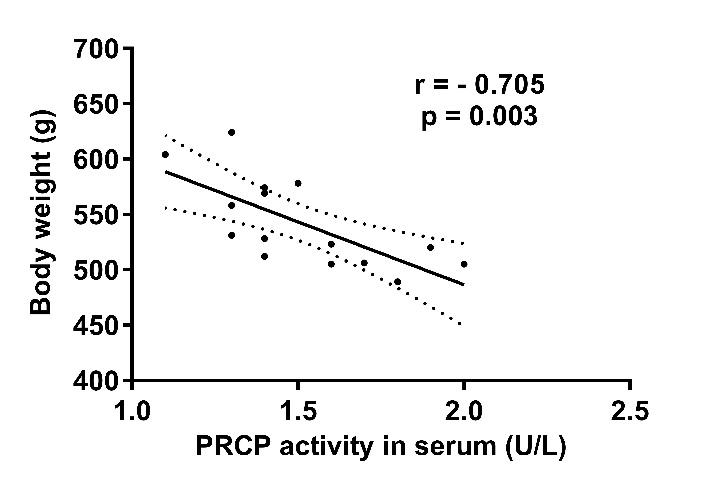


Figure 4.2 Inverse correlation between serum PRCP activity and body weight in rats with metabolic syndrome (n=16).
